# Coinhibitory Effects of Resveratrol- and Protopanaxadiol-Enriched Rice Seed Extracts Against Melanogenic Activities in Melan-a Cells

**DOI:** 10.3390/plants13233385

**Published:** 2024-12-01

**Authors:** Chaiwat Monmai, Yong-In Kuk, So-Hyeon Baek

**Affiliations:** 1 Department of Agriculture Life Science, Sunchon National University, Suncheon 59722, Republic of Korea; bbuayy@gmail.com; 2 Department of Oriental Medicine Resources, Sunchon National University, Suncheon 57922, Republic of Korea; yikuk@scnu.ac.kr

**Keywords:** synergistic effect, antioxidant, hyperpigmentation, antimelanogenic, NF-κB, MAPK

## Abstract

In the current study, we aimed to evaluate the combined antimelanogenic effects of resveratrol- and protopanaxadiol (PPD)-enriched rice seed extracts (DJ526 and DJ-PPD) in melan-a cells. The treatment antioxidant capacity was evaluated using the ABTS radical scavenging method. TR_3 (70% [wight (*w*)/*w*] of DJ526 and 30% [*w*/*w*] of DJ-PPD) markedly increased the antioxidant activity at a level similar to that of DJ526 and DJ-PPD alone. The antimelanogenic activities in melan-a cells were evaluated after co-culturing of treatments at the concentration of 100 μg/mL. The in vitro melan-a cell experiment showed that treatment with the DJ526 and DJ-PPD mixture significantly reduced the cellular tyrosinase activity and melanin content; suppressed the expression of melanogenesis-related genes and proteins; decreased the number and size of melanin-containing cells; upregulated phosphorylated extracellular signal-regulated kinase 1/2 and protein kinase B expression levels; and suppressed the expression of p-p38 MAPK. These results show that DJ-PPD does not interfere with the antioxidant and antimelanogeneic activities of DJ526 but enhances the antioxidant and antimelanogeneic activities of DJ526. These findings indicate the potential of resveratrol- and PPD-enriched rice seeds as novel agents for controlling hyperpigmentation.

## 1. Introduction

Melanin is produced by melanocytes through a process called melanogenesis [1]. The microphthalmia-associated transcription factor (MITF) is the melanogenesis-associated transcription factor, which controls the production of several enzymes, including tyrosinase-related protein (TRP)-1, TRP-2, and tyrosinase [2,3,4]. The regulation of MITF is related to the activation of mitogen-activated protein kinases (MAPKs) and protein kinase B (Akt) signaling pathways, including phosphorylated (p)-p38 MAPK, p-ERK 1/2, and p-Akt [5,6,7]. After melanin synthesis, it is transferred to keratinocytes to determine the color of the body skin and hair (main pigments in the human skin) [8,9]. Melanin can be divided into five types based on the various chemical precursors used in their biosynthesis, including eumelanin, pheomelanin, neuromelanin, allomelanin, and pyomelanin [10]. Generally, skin and hair pigments are associated with eumelanin and pheomelanin [11]. Black/brown pigmentation is associated with eumelanin, and lighter pigment skin is associated with pheomelanin [12,13]. Naturally, eumelanin and pheomelanin are always mixed at different ratios depending on the pigment origin and exposure to environmental factors [14,15]. The production of melanin plays an important role in protection from harmful solar UV radiation [16]. Pigmentation is also an important contributing factor to the skin and hair appearance [17]. Although the production of melanin protects the skin, excess production of melanin can cause hyperpigmentation, darker spots on the skin, melasma, and skin discoloration [18,19,20,21,22].

Resveratrol (cis- and trans-3,4′,5-trihydroxystilbene), a polyphenolic compound, has been found in plants and foods such as grapes (*Vitis vinifera*; 0.16–3.54 µg/g), blueberry (*Vaccinium myrtillus*; ≈0.032 µg/g), peanuts (*Arachis hypogaea*; 0.02–1.92 µg/g), jackfruits (*Artocarpus heterophyllus*; ≈3.56 µg/g), plums (*Prunus domestica*; 0.10–6.20 µg/g), and red wine (<0.10–2.10 µg/mL) [23,24,25,26,27,28,29,30,31]. Several studies have reported the potential of resveratrol and its derivative as human health agents related to anti-inflammatory [32,33,34,35], antioxidant [32,36], anticancer [37,38], antifungal [39], antibacterial [39], and antiaging effects [40,41,42]. Treatment with 0.125–4 µg/mL of resveratrol significantly increased the anti-inflammatory activities in human retinal pigment epithelial cells [35]. Chen et al. [34] synthesized the novel resveratrol-based flavonol derivatives and found that 10 μM of 2-(2,4-dimethoxy-6-(4-methoxystyryl)phenyl)-3-hydroxy-4H-chromen-4-one gives the most anti-inflammatory activities in RAW264.7 cells. Clément et al. [43] demonstrated that the death of promyeloblasts (HL-60: acute promyelocytic leukemia) and human breast cancer cells (T47D) was induced by resveratrol (4–32 μM). In addition, the oral administration of resveratrol (10 mg/kg/day) markedly inhibited reactive oxygen species in fructose-induced hypertensive rats [44]. Paulo et al. [45] reported the antibacterial effect of resveratrol, such as *Bacillus cereus* [American type culture collection (ATCC) 11778; minimum inhibitory concentration (MIC) is 50 μg/mL], *Staphylococcus aureus* (ATCC 25923; MIC is 100 μg/mL), three clinically isolated *Staphylococcus aureus* strains (MIC is 100–200 μg/mL), *Enterococcus faecalis* (ATCC 29212; MIC is 100 μg/mL), *Escherichia coli* (ATCC 25922; MIC is >400 μg/mL), *Klebsiella pneumoniae* (ATCC 13883; MIC is >400 μg/mL), and *Pseudomonas aeruginosa* (ATCC 27853; MIC is >400 μg/mL). The minimum inhibitory concentration of resveratrol on the antifungal had also been reported, including *Trichophyton mentagrophytes* (ATCC 18748; MIC is 25–50 μg/mL) [46], *T. tonsurans* (ATCC 28942; MIC is 25–50 μg/mL) [46], *T. rubrum* (ATCC 18762; MIC is 25–50 μg/mL) [46], *Candida albicans* (TIMM 1768; MIC is 20 μg/mL) [47], and *Saccharomyces cerevisiae* (Korean collection for type cultures 7296; MIC is 10–20 μg/mL) [47]. Additionally, treatment with resveratrol effectively reduced melanin production in B16 murine melanoma cells (10 μM), melanin production in human epidermal melanocytes (10 μM), and melanin production in a reconstituted human skin model (125 μM) [48]. Resveratrol-enriched rice (DJ526) was previously generated in Dongjin rice through genetic engineering by transferring the *Arachis hypogaea stilbene synthase* [49]. The contents of resveratrol and piceid in DJ526 were measured using high-performance liquid chromatography analysis [50]. DJ526 has been demonstrated to have anti-inflammatory activities through the inhibition of NF-κB and MAPK pathway activation, resulting in the downregulation of inflammatory-related cytokines and mediators in lipopolysaccharide (LPS)-stimulated RAW264.7 cells [50]. Treatment with DJ526 has been shown to significantly suppress adipogenic transcription factors, adipogenic genes, and inflammatory-associated molecules in 3T3-L1 cells [51]. Additionally, the resveratrol and piceid content in DJ526 rice seeds has been shown to be improved by callus induction, and the DJ5266 rice callus preserves the anti-inflammatory properties in LPS-induced RAW264.7 cells [52].

Compounds (crude and purified) extracted from ginseng have been widely used for pharmacological benefits [53], which relate to the anti-inflammatory [54] (treatment with 20(*S*)-PPD-type saponins extracted from *P. notoginseng* leaves inhibit LPS-induced NO release in a dose-dependent manner at 1, 10, and 25 μM), immunomodulatory [55] (treatment with isolated polysaccharide fraction of *P. ginseng* at 100 μg/mL increases the immune-related cytokine levels in isolated macrophages), neuroprotective [56,57] (ginseng root extract-treated astrocytes (1 mg/mL) significantly reduced oxidative stress), antifungal [58] [ginseng extract concentrations at which fungi proliferation was reduced by 80% are 16 (*Candida albicans*), 64 (*Cryptococcus neoformans*), and >64 (*Trichophyton rubrum*) μg/mL], and anticancer [58] [in vitro anticancer activity of the extracts of ginseng on the human gastric adenocarcinoma (MKN45; IC_50_ is 18.7 μg/mL), LoVo cells (IC_50_ is 9.2 μg/mL), and human liver cancer cell (HepG2; IC_50_ is 72.18 μg/mL)] effects. One of the main active compounds in ginseng is ginsenoside, which can be divided into two major types, protopanaxadiol (PPD) and protopanaxatriol glycosides [59,60]. Protopanaxadiol-enriched rice (DJ-PPD) was developed by introducing *Panax ginseng* dammarenediol-II synthase and protopanaxadiol synthase genes to DJ rice [61] and the production of protopanaxadiol (PPD) in the seeds was measured using liquid chromatography–mass spectrometry [62]. DJ-PPD (10–100 μg/mL) has several benefits for health, including immune enhancement via activating of NF-κB and MAPK signaling pathways in RAW264.7 cells, which led to an increase in NO production, PGE_2_ production, and immune-related cytokine expression [62,63], anti-inflammatory effects, by inhibiting the activation of NF-κB and MAPK signaling pathways in LPS-stimulated RAW264.7 cells [62,63], and antiadipogenic activities, by suppressing the activation of adipogenic transcription factors at the mRNA and protein levels, resulting in a reduction in adipogenic-related mRNA, cell population in the G2/M phase, and lipid accumulation in 3T3-L1 cells [64].

DJ526 and DJ-PPD exhibit oxidative and melanogenesis properties in melan-a cells [65,66]. The melanogenesis transcription factor is suppressed in the cells treated with DJ526 and DJ-PPD, which leads to a reduction in melanogenesis-related gene expression levels, the number of melanin-containing cells, cell size, and cellular tyrosinase activity. Several studies have demonstrated the antimelanogenic activity by a combination of plant extracts. Ssanghwa-tang, traditional herbal medicine, consists of nine medicinal herbs, which effectively suppressed the melanin synthesis in B16F10 cells [67]. The combination of chia (*Salvia hispanica*) seed and pomegranate (*Punica granatum*) fruit extracts markedly downregulated the expression level of key melanogenic genes in melan-a cells [68]. Kim et al. [69] reported the synergistic antimelanogenic effects of commercial ginseng (*Panax ginseng*: Ginseng Radix Alba) and mulberry (*Morus alba*: Mori Radicis Cortex) extracts in B16 cells. Therefore, in the present study, we aimed to investigate the cotreatment effect between DJ526 and DJ-PPD in the difference ratio of mixture (*w*/*w*) on the antioxidant and antimelanogenic activities in melan-a cells.

## 2. Results

### 2.1. ABTS Radical Scavenging Activity of DJ526 and DJ-PPD in Combination

**Figure 1:**
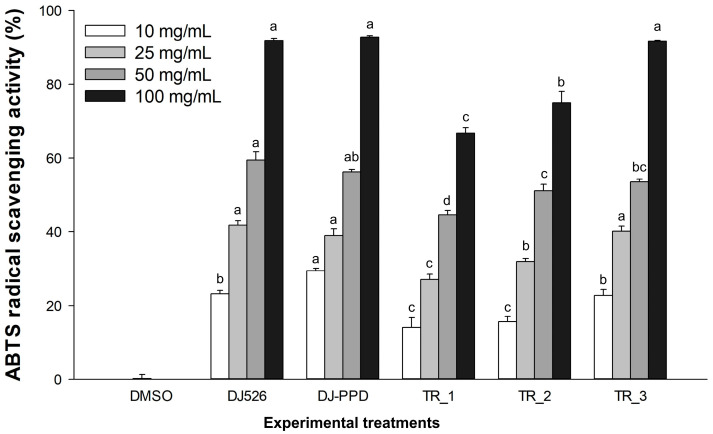
Antioxidant activity of DJ526 and DJ-PPD in combination. The data are presented as the mean ± standard deviation (*n* = 3). Lowercase letters represent the significant differences in ABTS radical scavenging activity between the treatments at the same concentration at *p* < 0.05. The letter “a” represents the reference value, “b” represents significantly lower value than “a” (*p* < 0.05), and “c” represents significantly lower value than “b” (*p* < 0.05).

The antioxidant effect of DJ526 combined with DJ-PPD was evaluated through the ABTS radical scavenging activity comparison with that of the DMSO group. At the same concentration, DJ526 or DJ-PPD alone exhibited the highest ABTS radical scavenging activity (Figure 1). Among the treatments, the highest ABTS radical scavenging activity was observed in the TR_3 group [30% (*w*/*w*) of DJ-PPD and 70% (*w*/*w*) of DJ526]. At the concentration of 100 mg/mL, TR_3 significantly inhibited the ABTS radical scavenging activity at the same level as that of DJ526 and DJ-PPD. DJ526, DJ-PPD, and TR_3 at concentrations of 40.75 ± 0.66, 40.11 ± 0.59, and 43.52 ± 0.29 mg/mL, respectively, inhibited the ABTS radical scavenging activity by 50% (IC_50_), which exhibited the lowest IC_50_ level (*p* < 0.05) among all the treatments (Table 1). The IC_50_ levels of TR_1 and TR_2 were 67.06 ± 1.36 and 56.65 ± 2.78 mg/mL, respectively. Therefore, TR_3 exhibited higher antioxidant activity (ABTS radical scavenging activity) than TR_1 and TR_2, which showed no significant difference compared to DJ526 and DJ-PPD alone.

### 2.2. Cell Viability Effect of DJ526 and DJ-PPD in Combination

**Figure 2:**
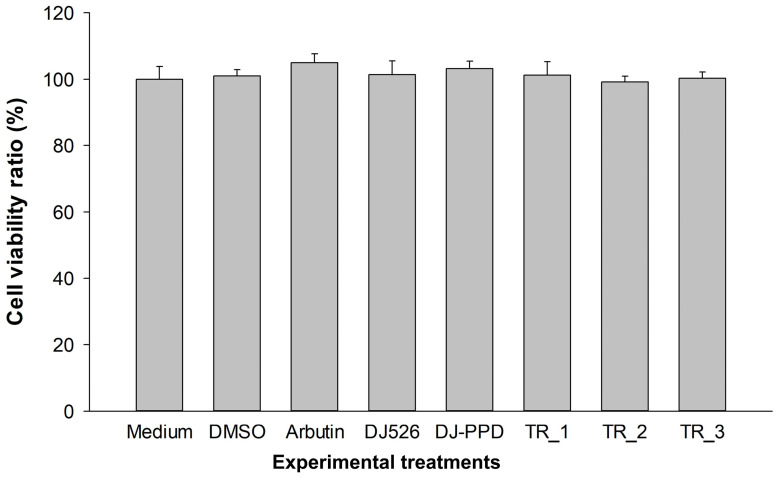
Effect of combined DJ526 and DJ-PPD on the viability of melan-a cells. The concentrations of DMSO and the treatments were 0.1% (*v*/*v*) and 100 µg/mL, respectively. The data are presented as the mean ± standard deviation (*n* = 3). The significant difference was determined at *p* < 0.05 compared to the medium group.

The cytotoxicity of the treatments was measured using an EZ-Cytox cell viability kit. Figure 2 shows that the treatment of arbutin, DJ526, DJ-PPD, TR_1, TR_2, and TR_3 at the concentration of 100 µg/mL did not cause cell cytotoxicity on melan-a cells. Compared to the medium-treated cells, the DJ526-, DJ-PPD-, TR_1-, TR_2, and TR_3-treated cells showed similar levels of cell viability (*p* < 0.05). Therefore, treatments at a concentration of 100 µg/mL were used in the in vitro melan-a cell experiments.

### 2.3. Melanin Content and Cellular Tyrosinase Activity of DJ526 and DJ-PPD in Combination

**Figure 3:**
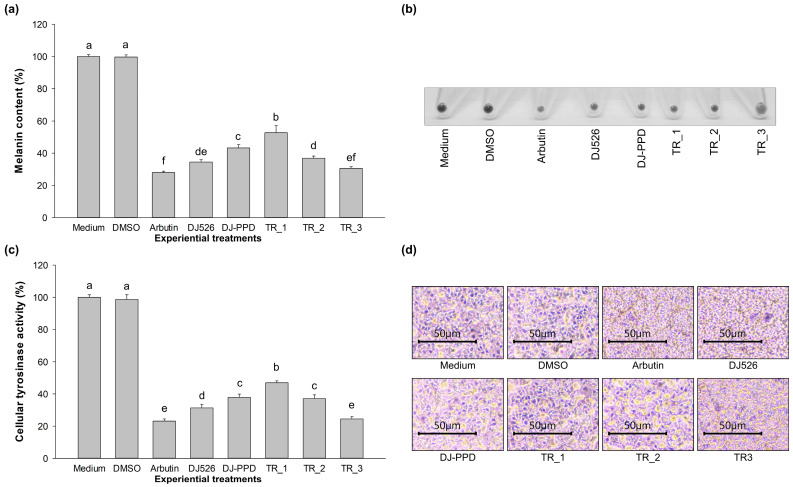
Effects of the combination of DJ526 and DJ-PPD on the melanin content and cellular tyrosinase activity in melan-a cells. (**a**) Melanin content (%) compared to the medium group, (**b**) representative of cell pellet collection, (**c**) cellular tyrosinase activity (%) compared to the medium group, and (**d**) representative of L-DOPA staining on melan-a cells. The concentrations of DMSO and the treatments were 0.1% (*v*/*v*) and 100 µg/mL, respectively. The data are presented as the mean ± standard deviation (*n* = 3). Significant differences among the treatments are indicated using the different letters (a, b, c, d, e, and f) at *p* < 0.05. The letter “a” represents the reference value, “b” represents significantly lower value than “a” (*p* < 0.05), “c” represents significantly lower value than “b” (*p* < 0.05), “d” represents significantly lower value than “c” (*p* < 0.05), “e” represents significantly lower value than “d” (*p* < 0.05), “f” represents significantly lower value than “e” (*p* < 0.05), and the same letter represents no significant difference (*p* < 0.05).

The melanin content was measured in the treated melan-a cells. Arbutin, which was used as a positive control, significantly reduced the melanin content compared to the medium- and DMSO-treated cells (Figure 3a,b). Treatments with DJ526, DJ-PPD and the mixture of DJ526 and DJ-PPD significantly decreased the melanin content when compared to the medium group. Treatment with TR_3 effectively inhibited the melanin content in melan-a cells at a level similar to that of arbutin (*p* < 0.05). Cellular tyrosinase was evaluated in the melan-a cells after culture with the treatments. Figure 3c shows that treatment with arbutin and TR_3 resulted in the highest reduction in cellular tyrosinase activity. The inhibition of cellular tyrosinase activity by TR_3 was markedly higher when compared with that by DJ526 or DJ-PPD alone. In addition, the melanin-containing cells from each treatment were stained with L-DOPA. The dark spots representing the melanin staining of the melanin-containing cells were normally observed in the medium-treated cells (Figure 3d). Treatment with arbutin, DJ526, DJ-PPD, and the mixture of DJ526 and DJ-PPD observably reduced the dark spots of L-DOPA staining. These results indicate that treatment with TR_3 significantly reduced the cellular tyrosinase activity when compared to DJ526 or DJ-PPD treatment alone and markedly decreased the melanin content compared to DJ-PPD.

### 2.4. Melonogenesis-Related Gene Expression Effect of DJ526 and DJ-PPD in Combination

**Figure 4:**
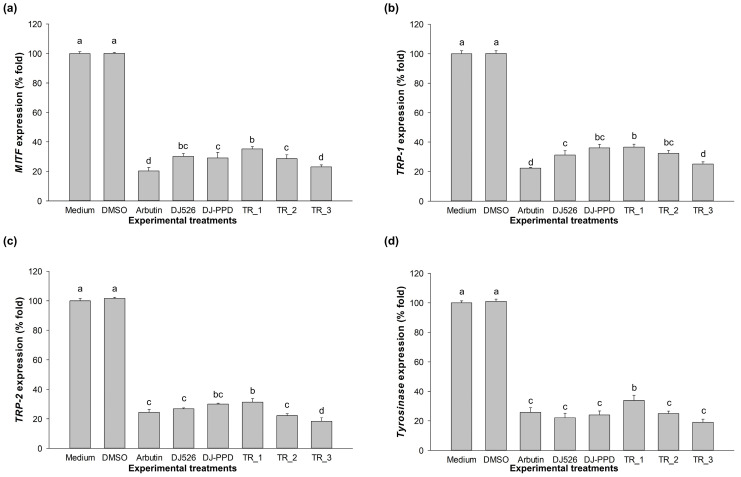
Effects of the combination of DJ526 and DJ-PPD on melanogenesis-related gene expression levels. Effect on the (**a**) *MITF* expression level, (**b**) *TRP-1* expression level, (**c**) *TRP-2* expression level, and (**d**) *tyrosinase* expression level. The concentrations of DMSO and the treatments were 0.1% (*v*/*v*) and 100 µg/mL, respectively. The data are presented as the mean ± standard deviation (*n* = 3). Significant differences in gene expression levels among the treatments are indicated using the different letters (a, b, c, and d) at *p* < 0.05. The letter “a” represents the reference value, “b” represents significantly lower value than “a” (*p* < 0.05), “c” represents significantly lower value than “b” (*p* < 0.05), “d” represents significantly lower value than “c” (*p* < 0.05), and the same letter represents no significant difference (*p* < 0.05).

The expression levels of melanogenesis-associated genes (*MITF*, *TRP-1*, *TRP-2*, and *tyrosinase*) in the medium-treated cells were set as 100% expression (Figure 4). Treatment with arbutin, DJ526, DJ-PPD, TR_1, TR_2, and TR_3 at 100 µg/mL markedly downregulated the expression levels of *MITF*, *TRP-1*, *TRP-2*, and *tyrosinase* when compared to the medium group. The gene expression levels of melanogenesis-related genes in cells treated with 0.1% DMSO did not show a significant difference (*p* < 0.05) when compared to the medium group. For the positive control, arbutin-treated cells exhibited the lowest expression of *MITF*, *TRP-1*, and *tyrosinase* among all the treatments. Treatment with TR_3 significantly downregulated the expression of *MITF*, *TRP-1*, and *tyrosinase* at levels similar to those in the arbutin group. Additionally, TR_3-treated cells remarkedly suppressed the expression of *TRP-2* when compared to arbutin. Interestingly, treatment with TR_3 significantly inhibited the expression of *MITF*, *TRP-1*, and *TRP-2* when compared with DJ526 or DJ-PPD alone. These results indicate that treatment with DJ526, DJ-PPD, and their mixture effectively reduced the expression of melanogenesis-related genes in melan-a cells.

### 2.5. Melanogenesis-Associated Protein Expression Levels of DJ526 and DJ-PPD in Combination

**Figure 5:**
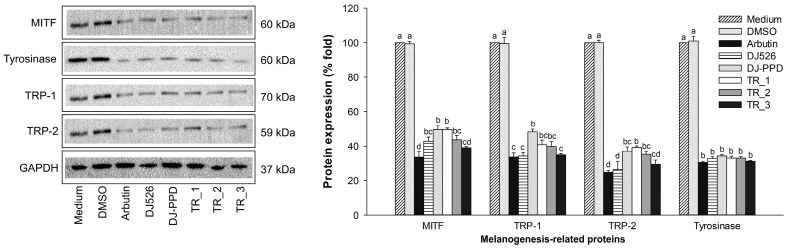
Effect of DJ526 and DJ-PPD in combination on melanogenesis-related protein expression levels. The concentrations of DMSO and the treatments were 0.1% (*v*/*v*) and 100 µg/mL, respectively. The data are presented as the mean ± standard deviation. Significant differences in protein expression levels among the treatments are indicated using the different letters (a, b, c, and d) at *p* < 0.05. The letter “a” represents the reference value, “b” represents significantly lower value than “a” (*p* < 0.05), “c” represents significantly lower value than “b” (*p* < 0.05), “d” represents significantly lower value than “c” (*p* < 0.05), and the same letter represents no significant difference (*p* < 0.05).

The expression levels of proteins associated with melanogenesis were evaluated using blot analysis (Figure 5). The highest expression of MITF, TRP-1, TRP-2, and tyrosinase protein was observed in medium- and DMSO-treated cells. Treatment with 100 µg/mL of arbutin, DJ526, DJ-PPD, TR_1, TR_2, and TR_3 significantly decreased the expression of melanogenesis-associated protein expressions (*p* < 0.05). Arbutin and TR_3 exhibited the highest suppression of MITF, TRP-1, TRP-2, and tyrosinase expression levels. These results indicate that TR_3 markedly suppressed melanogenesis by inhibiting the melanogenesis-related protein expression levels.

### 2.6. Morphological Appearance Effect of DJ526 and DJ-PPD in Combination

**Figure 6:**
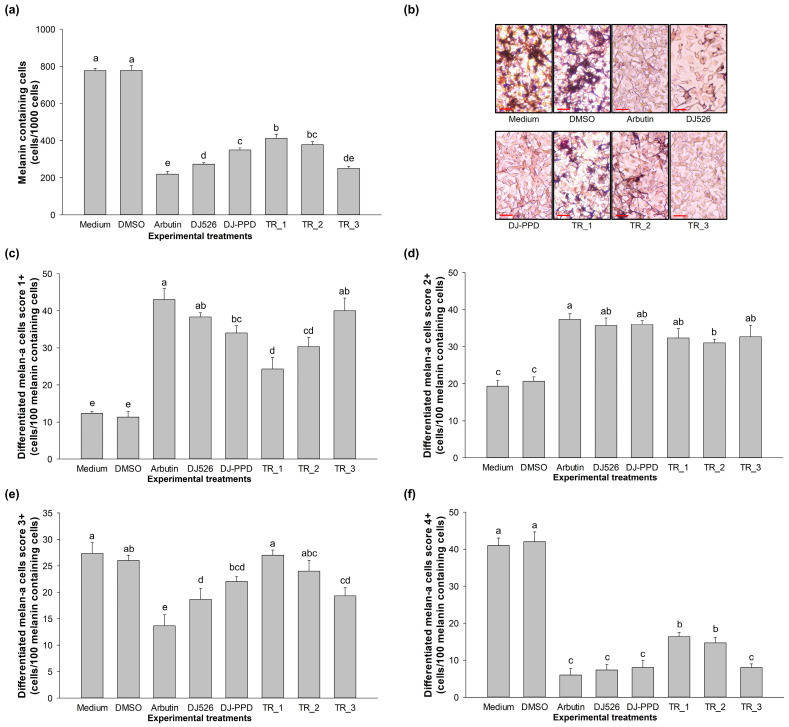
Effect of the combination of DJ526 and DJ-PPD on the morphology and differentiation of melan-a cells. Effect on (**a**) melanin-containing cells in the total of 1000 cells, (**b**) Fontana–Masson staining with scale bar at 50 μm (−), (**c**) melan-a cell differentiation score 1+, (**d**) melan-a cell differentiation score 2+, (**e**) melan-a cell differentiation score 3+, (**f**) melan-a cell differentiation score 4+. The concentrations of DMSO and the treatments were 0.1% (*v*/*v*) and 100 µg/mL, respectively. The data are presented as the mean ± standard deviation (*n* = 3). Significant differences in melanin-containing cells and melan-a cell differentiation score levels among the treatments are indicated using the different letters (a, b, c, d, and e) at *p* < 0.05. The letter “a” represents the reference value, “b” represents significantly lower value than “a” (*p* < 0.05), “c” represents significantly lower value than “b” (*p* < 0.05), “d” represents significantly lower value than “c” (*p* < 0.05), “e” represents significantly lower value than “d” (*p* < 0.05), and the same letter represents no significant difference (*p* < 0.05).

The cells were stained with the Fontana–Masson staining assay to evaluate the melanin-containing cell number and morphological appearance. At the total of 1000 cells, the average number of melanin-containing cells of the medium and DMSO group are 777.33 ± 12.10 and 779.00 ± 24.58 cells, respectively (Figure 6a). Treatment with DJ526 and DJ-PPD significantly decreased the number of melanin-containing cells (273.33 ± 08 and 348.67 ± 13.01 cells) compared to the medium group (*p* < 0.05). In comparison with DJ-PPD, treatment with TR_3 (251.00 ± 8.19 cells) significantly reduced the number of melanin-containing cells, which is similar to the results of the cells treated with arbutin (218.67 ± 16.50 cells) (*p* < 0.05).

The melanin-containing cells were randomly counted at a total of 100 cells and then divided into four groups (Figure 6b), as described in Figure S3 and Table S2. For the nontreatment (medium) group, the average population of melan-a cells in each differentiated score was 4+ (41.00 ± 2.00 cells), 3+ (27.33 ± 2.08 cells), 2+ (19.33 ± 1.53 cells), and 1+ (12.33 ± 0.58 cells) (Figure 6c–f). These results were similar to the DMSO-treated cell group, which showed the most cell population in the 4+ differentiated score (42.00 ± 1.00 cells). However, treatments with arbutin, DJ526, DJ-PPD, TR_1, TR_2, and TR_3 showed a difference in the cell population patent when compared to the medium group. Most of the arbutin-, DJ526-, and TR_3-treated cells were divided into the 1+ differentiated score (43.00 ± 3.00, 38.33 ± 1.15, and 40.00 ± 3.46 cells, respectively). For DJ-PPD, TR_1, and TR_2, most of the population cells were divided into the 2+ differentiated score (36.00 ± 1.00, 32.33 ± 2.52, and 31.00 ± 1.00 cells, respectively). These results indicate that treatment with TR_3 significantly reduced the number of melanin-containing cells, decreased the population of 4+ differentiated cells, and increased the population of 1+ differentiated cells.

### 2.7. MAPK and PI3K/Akt Signaling Pathway-Associated Protein Expression Levels of DJ526 and DJ-PPD in Combination

**Figure 7:**
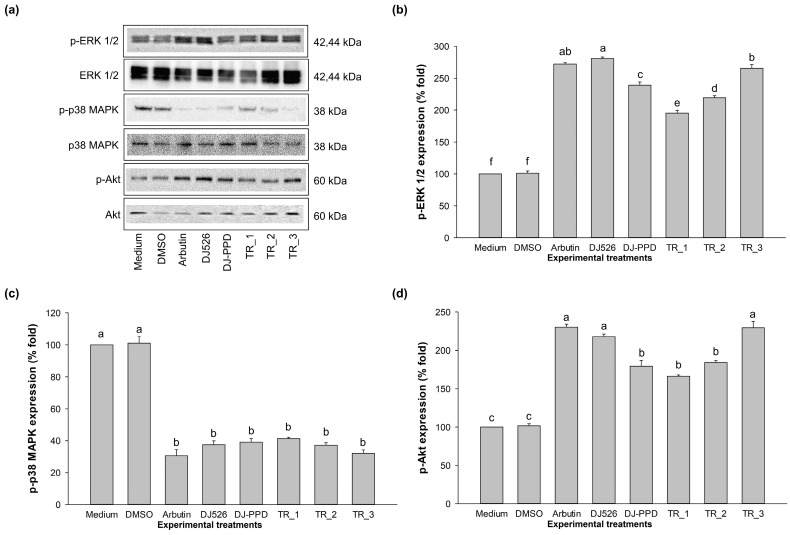
Effect of the combination of DJ526 and DJ-PPD on the MAPK and PI3K/Akt signaling pathways. (**a**) Blot analysis, (**b**) p-ERK 1/2 expression, (**c**) p-p38 MAPK expression, and (**d**) p-Akt expression. The data are presented as the mean ± standard deviation. Significant differences in the protein expression levels among the treatments are indicated using the different letters (a, b, c, d, e, and f) at *p* < 0.05. The letter “a” represents the reference value, “b” represents significantly lower value than “a” (*p* < 0.05), “c” represents significantly lower value than “b” (*p* < 0.05), “d” represents significantly lower value than “c” (*p* < 0.05), “e” represents significantly lower value than “d” (*p* < 0.05), “f” represents significantly lower value than “e” (*p* < 0.05), and the same letter represents no significant difference (*p* < 0.05).

The expression of p-ERK 1/2, p-p38 MAPK, and p-Akt protein has been reported to be related to the regulation of MITF protein. Figure 7 shows that treatment with arbutin, DJ526, DJ-PPD, TR_1, TR_2, and TR_3 significantly increased the expression levels of p-ERK 1/2 and p-Akt and decreased the expression level of p-p38 MAPK when compared to the nontreatment group (*p* < 0.05). In comparison with arbutin, the regulation of p-ERK 1/2, p-p38 MAPK, and p-Akt exhibited similar levels in the cells treated with DJ526 and TR_3. These results indicate that treatment with TR_3 at the concentration of 100 µg/mL effectively regulated the expression of p-ERK 1/2, p-p38 MAPK, and p-Akt, which is similar to the results of the cells treated with the positive control.

## 3. Discussion

In this study, the resveratrol-enriched rice seed extract and PPD-enriched rice seed extract were evaluated for their cotreatment effects on antioxidant and antimelanogenesis activities in melan-a cells. At 100 mg/mL, the mixture of DJ526 and DJ-PPD significantly increased the ABTS radical scavenging activity in a dose-dependent manner of the DJ526 amount in the mixture (Figure 1 and Table 1). The antioxidant (ABTS radical scavenging) activity of the mixture was correlated with the DJ526 content in the mixture (Pearson’s correlation coefficient = 0.9599, *p* < 0.01). Additionally, mixing DJ-PPD (30% *w*/*w*) with DJ526 (70% *w*/*w*) markedly increased the activity of ABTS radical scavenging, which exerted a similar level as that by DJ526 and DJ-PPD alone. The effect of cotreatment by resveratrol and PPD on the antioxidant activity was also reported by Huangfu et al. [70]. The nanoparticle complex between resveratrol and 20(*S*)-PPD was generated and the antioxidant was investigated by the ABTS and reactive oxygen species (ROS) method. Huangfu et al. [70] suggested that PPD plays a synergistic antioxidant role with resveratrol by enhancing the ROS scavenging rate when compared to that of resveratrol alone. Zhang et al. [71] demonstrated an additive effect in ABTS radical scavenging capacity, which was observed for resveratrol mixture with α-tocopherol at vitamin concentrations below 0.2 mg/mL. Murugesan et al. [72] evaluated and suggested the synergistic antioxidant efficacy of quercetin and resveratrol in combination using four kinds of antioxidant analysis methods, including, Diphenyl-2-picrylhydrazyl, ABTS radical, and superoxide anion scavenging activity assay. Hashemi et al. [73] reported that the radical scavenging evaluations of 2,2-Diphenyl-1-picrylhydrazyl and ABTS+ showed better antioxidant activities of the films containing resveratrol than the films containing zataria multiflora boiss essential oil and butylated hydroxytoluene.

MITF is the transcription factor involved in the process of melanin synthesis [74]. The activation of MITF results in the upregulation of melanogenesis-related genes and enzymes, including TRP-1, TRP-2, and tyrosinase [75]. To synthesize the eumelanin (common form of melanin), tyrosinase is an important enzyme for hydroxylating the L-tyrosine to L-dihydroxyphenylalanine (L-DOPA) and oxidizing the L-DOPA to DOPA-quinone [76]. TRP-1 and TRP-2 are the other two enzymes required during the melanogenesis process [77,78]. The regulation of MITF has been reported to be associated with the activation of the MAPK and PI3K/Akt signaling pathways [7,79]. The activation of p-ERK 1/2 and p-Akt inhibit MITF activity by increasing MITF ubiquitination and degradation [80]. However, the degradation of MITF is suppressed by the activation of p-p38 MAPK [81].

Our results demonstrate the antimelanogenesis effects of the combination of DJ526 and DJ-PPD on melan-a cells. The production of the melanogenesis-associated transcription factor (at both the gene and protein levels), MITF, significantly decreased when the cells were treated with TR_1, TR_2, and TR_3 (Figure 4 and Figure 5). Additionally, the expression of the MITF promoter, p-p38 MAPK, markedly decreased in the TR_1-, TR_2-, and TR_3-treated cells (Figure 7c). Conversely, treatment with TR_1, TR_2, and TR_3 significantly promoted the MITF degradation inducers p-ERK 1/2 and p-Akt (Figure 7b,d). These results were supported by the report of Bhat et al. [82], which demonstrated the downregulation of MITF, TRP-1, TRP-2, and tyrosinase expression by inhibiting the activation of p-p38 MAPK. Additionally, melanogenesis is inhibited by the activation of p-ERK 1/2 and p-Akt, which is related to the increase in MITF degradation [83]. The downregulation of MITF resulted in a reduction in TRP-1, TRP-2, and tyrosinase at both the mRNA and protein levels. The decrease in tyrosinase activity was confirmed using L-DOPA, the substrate of tyrosinase in melanogenesis. Interestingly, treatment with DJ526 together with DJ-PPD (TR_3) effectively decreased the cellular tyrosinase activity compared to that by DJ526 and DJ-PPD alone (Figure 3c,d). In addition to the inhibition effects on the expression of melanogenesis-related proteins (MITF, TRP-1, TRP-2, and tyrosinase) and cellular tyrosinase activity, the combination of DJ526 and DJ-PPD effectively decreased the number of melanin-containing cells (Figure 6a), cell size, contribution of melanin in cells (Figure 6c–f), and melanin accumulation (Figure 3a). The synergistic effect of resveratrol and other compounds on the antioxidant and antimelanogenesis effects has been reported in several studies. Jin et al. [84] confirmed the synergistic effect of resveratrol and oxyresverartrol against the oxidative and melanogenic activities in human epidermal melanocytes. Kim et al. [85] reported the synergetic melanin synthesis inhibition effect of resveratrol and 4-n-butylresorcinol, but did not inhibit the production of melanin by resveratrol and 4-n-butylresorcinol individually. A preclinical study on the dorsal skin of Guinea pigs demonstrated the potential of trans-resveratrol combined with hydrophilic surfactants and oils as a skin-whitening agent [86]. Additionally, a cream containing resveratrol and 4-n-butylresorcinol was produced and shown to be effective and safe for the treatment of melasma in the patients [87]. Feng et al. [88] suggested that the combination with the five plant extracts (*Panax ginseng*, *Polygonatum cyrtonema*, *Epiphyllum oxypetalum*, *Nelumbo nucifera*, and *Osmanthus fragrans*) showed a stronger skin anti-aging effect compared to *Panax ginseng* alone. Additionally, the mixture of ginseng and mulberry tree extracts enhances the anti-melanogenic effect in B16F10 cells by reducing the tyrosinase activity, cellular tyrosinase activity, melanin secretion, number of dark spots, and pigmented cell areas [69]. Kim et al. [89] evaluated the skin-lightening synergistic effect of ginsenosides Rh1, Rg2, and *Hydrangea* extract. The results show that the combination of these mixture more effectively reduced the tyrosinase activity and suppressed the melanin synthesis than that by each component alone. In addition, the combination of ginsenosides Rh1, Rg2, and *Hydrangea* extract enhanced the permeability and efficacy of Rh1.

## 4. Materials and Methods

### 4.1. Experimental Treatments

DJ526 and DJ-PPD were extracted and the amount of resveratrol (piceid and resveratrol) or PPD contained in the seeds was measured previously [50,62]. These extracts were used to evaluate the in vitro antimelanogenesis in this study. The amount of resveratrol and piceid contained in the seeds of DJ526 was 2.605 ± 0.001 and 4.724 ± 0.020 µg/g dry weight (DW), respectively. The PPD amount in the DJ-PPD seeds was 7.28 ± 0.640 µg/g DW. The experimental treatments were assigned as DJ526 (100% *w*/*w* of DJ526), DJ-PPD (100% *w*/*w* of DJ-PPD), TR_1 (30% *w*/*w* of DJ526 combined with 70% *w*/*w* of DJ-PPD), TR_2 (50% *w*/*w* of DJ526 combined with 50% *w*/*w* of DJ-PPD), and TR_3 (70% *w*/*w* of DJ526 combined with 30% *w*/*w* of DJ-PPD). The amounts of piceid, resveratrol, and PPD in the treatment are shown in Table S1. The samples were prepared at concentrations of 10, 25, 50, and 100 mg/mL in DMSO for the ABTS radical scavenging activity investigation and a concentration of 100 mg/mL for the in vitro melan-a cell experiments.

### 4.2. ABTS Radical Scavenging Activity

The 2,2-Azino-bis-3-ethylbenzothiazoline-6-sulfonic acid (ABTS; Roche, Basel, Switzerland) solution was mixed with potassium persulfate solution at the ratio of 1:1 (*v*/*v*) at final concentrations of 7.0 and 2.4 mM, respectively. The mixture solution was incubated at room temperature for 16 h and protected from light. The working solution of ABTS^•+^ was prepared at an absorbance value of 0.70 ± 0.02 at 734 nm by dilution in absolute ethanol. The ABTS^•+^ was incubated with each treatment at a ratio of 100:1 (*v*/*v*) for 7 min in the dark. Distilled water served as the control. The incubated solution was measured for absorbance at 734 nm and ABTS radical scavenging activity was calculated using Formula (1).

(1) $$\mathrm{ABTS}\;\mathrm{radical}\;\mathrm{scavenging}\;\mathrm{activity}\;(\%)=\left(\frac{A_{734}\mathrm{of}\;\mathrm{control}-A_{734}\mathrm{of}\;\mathrm{treatment}}{A_{734}\mathrm{of}\;\mathrm{control}}\;\text{×}\;100\right)$$

where A_734_ represents the absorbance value at 734 nm.

The concentration of treatments required to inhibit the ABTS radical scavenging activity by 50% (IC_50_) was evaluated by plotting the graph between the ABTS radical scavenging activity value (X-axis) and treatment concentrations (Y-axis). The regression equation was generated and the x value in the regression equation was substituted with 50 to calculate the IC_50_ of each treatment. The representative standard curves of each treatment are shown in Figure S1.

### 4.3. Vitamin C Equivalent Antioxidant Capacity

The vitamin C equivalent antioxidant capacity (VCEAC) was evaluated using the standard curve plotting the ABTS radical scavenging activity against the concentration of ascorbic acid (Sigma-Aldrich, St. Louis, MO, USA). The VCEAC was calculated using Formula (2) according to the information in Figure S2.

(2) VCEAC (mg/g DW)=2.763x−20.024

where x represents the value of the ABTS radical scavenging activity.

### 4.4. Melan-a Cell Culture

The melan-a cells were cultured in a cell culture medium composed of fetal bovine serum (10% *v*/*v*; Gibco™, Thermo Fisher Scientific, Inc., Waltham, MA, USA), penicillin/streptomycin (1% *v*/*v*; Hyclone Laboratories, Inc., Logan, UT, USA), and 12-*O*-tetradecanoylphorbol-13-acetate (20 mM, TPA; Sigma-Aldrich) in RPMI-1640 medium (Gibco™). The cells were incubated at 37 °C under 5% CO_2_.

### 4.5. Cell Viability

Cells at a concentration of 2 × 10^4^ cells were seeded into each well of a 96-well plate. After 24 h of incubation, the cell culture medium was discarded. The treatments (DJ526, DJ-PPD, TR_1, TR_2, and TR_3) were prepared at 100 µg/mL in the cell culture medium and were added into the specified wells. Cell culture medium, 0.1% DMSO, and 100 µg/mL arbutin (Sigma-Aldrich) served as negative, model, and positive controls, respectively. The plate was incubated at 37 °C under 5% CO_2_ for 72 h. The culture medium was replaced with EZ-Cytox (DoGenBio, Seoul, Republic of Korea) working solution (diluted 1:10 *v*/*v* in 1× phosphate buffered saline (PBS)). The plate was incubated at 37 °C for 4 h. The optical density of the solution was measured at 450 nm using a SpectraMax^®^ ABS Plus Microplate Reader (Molecular Devices, San Jose, CA, USA). Formula (3) was used for the cell ability ratio calculation.

(3) $$\mathrm{Cell}\;\mathrm{viability}\;\mathrm{ratio}\;(\%)=\frac{A_{450}\mathrm{of}\;\mathrm{treatment}}{A_{450}\mathrm{of}\;\mathrm{negative}\;\mathrm{control}}\;\text{×}\;100$$

where A_450_ represents the absorbance at 450 nm.

### 4.6. Melanin Content and Cellular Tyrosinase Activity

The treated cells were collected using trypsin-EDTA (Welgene, Gyeongsangbuk, Republic of Korea) and centrifuged at 1200 rpm for 3 min. The cells of each treatment were divided into two groups for melanin content and cellular tyrosinase activity evaluation. First, cells (1 × 10^5^ cells) were disrupted by incubation with 10% (*v*/*v*) DMSO in 1 N NaOH solution at 80 °C for 1 h. The optical density of the solution was measured at 405 nm and the melanin content was calculated using Formula (4).

(4) $$\mathrm{Melanin}\;\mathrm{content}\;(\%)=\frac{A_{405\;}\mathrm{of}\;\mathrm{treatment}}{A_{405\;}\mathrm{of}\;\mathrm{negative}\;\mathrm{control}}\;\text{×}\;100$$

where A_405_ represents the absorbance at 405 nm.

Second, the cell pellets were lysed with 1% (*v*/*v*) of Triton X-100 in 0.1 M sodium phosphate (pH 6.8) at room temperature for 30 min. The supernatant was collected by centrifugation at 13,000 rpm and 4 °C for 30 min. The concentration of protein was measured using a Bradford solution. Protein (40 µg) from each treatment was adjusted in a total of 80 µL with lysis buffer. The protein was mixed with 20 µL of L-DOPA (Sigma-Aldrich) (2 mg/mL) and the tubes were incubated at 37 °C for 1 h. The absorbance of the solution was measured at 475 nm, and the cellular tyrosinase activity was calculated using Formula (5).

(5) $$\mathrm{Cellular}\;\mathrm{tyrosinase}\;\mathrm{activity}\;(\%)=\left(\frac{{A_{475\;}\mathrm{of}\;\mathrm{medium}\;\mathrm{group}-A}_{475\;}\mathrm{of}\;\mathrm{treatment}\;\mathrm{group}}{A_{475\;}\mathrm{of}\;\mathrm{medium}\;\mathrm{group}}\right)\;\text{×}\;100$$

where A_475_ represents the absorbance at 475 nm.

### 4.7. L-DOPA Staining

The cells were seeded into a 96-well plate at a density of 2 × 10^4^ cells/well and the plate was incubated at 37 °C with 5% CO_2_ for 24 h. The medium was replaced with 100 µg/mL of each treatment prepared in the cell culture medium. The plate was incubated under the same conditions for a further 72 h. After discarding the cell culture medium, the cells were washed with ice-cold 1× PBS twice. The cells were fixed with 10% formaldehyde for 20 min at room temperature. After being fixed, the cells were washed with 1× PBS. Then, L-DOPA at a concentration of 2 mg/mL (100 µL) was added into each well and the plate was incubated at 37 °C. After 3 h of incubation, the supernatant was discarded and the cells were washed three times with 1× PBS. The stained cells were observed under an IM-3 series microscope (Optika, Bergamo, Italy).

### 4.8. RNA Isolation and cDNA Synthesis

Total RNA was extracted using TRI reagent™ (Invitrogen, Waltham, MA, USA) as previously described [66]. The quality and quantity of the isolated RNA were determined using a SpectraMax^®^ ABS plus microplate reader (Molecular Devices). The cDNA was synthesized from the good quality of RNA, which gave the ratio of the absorbance at 260 nm and 280 nm (A260:A280) and A260:A230 in the acceptable range of 1.8–2.0 (Table 2). Total RNA at 1000 ng was used for synthesizing the first strand of cDNA using a Power cDNA synthesis kit (Intron Biotechnology, Seongnam-si, Republic of Korea). The cDNA was prepared at 5 ng/µL in nuclease-free water (based on the RNA concentration).

### 4.9. Gene Expression Evaluation

The melanogenesis-related genes were measured for expression levels using a CFX connect Real-Time PCR system (Bio-Rad, Hercules, CA, USA). The reaction for qPCR was prepared in a total of 20 µL of RealMOD™ Green W^2^ 2× qPCR mix (Intron Biotechnology) consisting of specific primer [65] (0.375 µM of forward primer and 0.375 µM of reverse primer) and cDNA template (5 ng). The qPCR was performed as described previously [66]. The relative gene expression levels were investigated with regard to GAPDH using CFX Maestro software version 1.1 (Bio-Rad).

### 4.10. Morphological Appearance

The morphological appearance was evaluated by staining with a Fontana–Masson kit (BIOGNOST, Ltd., Zagreb, Croatia) according to the manufacturer’s instructions. The stained cells were randomly counted, with a total of 1,000 cells. Then, 100 melanin-containing cells were categorized into four morphological appearance groups (Figure S3 and Table S2), as described by Rodboon et al. [90].

### 4.11. Western Blot Analysis

Total protein was extracted from the treated cells using 1% protease inhibitor (Bio-Medical Science Co., Ltd., Seoul, Republic of Korea) in RIPA buffer (GeneAll Biotechnology, Seoul, Republic of Korea) and quantified using Bradford solution as previously performed [66]. The extracted protein from each treatment was adjusted to a concentration of 2 µg/µL using lysis buffer (RIPA buffer containing 1% of protease inhibitor). Thirty micrograms of protein were separated by SDS-polyacrylamide gel electrophoresis and transferred onto a nitrocellulose membrane (Immobilon^®^-P transfer membrane, Merck, Millipore, Burlington, MA, USA). The blot analysis was performed as described previously [91]. The membrane was incubated with specific antibodies (Table 3) at 4 °C overnight. The blot signals were detected with Pierce ECL plus a Western blot substrate (Thermo Scientific™, Waltham, MA, USA). The signals were captured by the ChemiDoc image system (Bio-Rad) and the intensity of the bands was quantified using Image lab software version 6 (Bio-Rad). The relative expression of protein was calculated as fold changes (%) compared to the nontreated group (medium).

### 4.12. Statistical Analysis

Statistical analyses were performed using Statistix (version 8.1; Statistix, Tallahassee, FL, USA). The data analysis was performed using a one-way analysis of variance followed by post hoc Duncan’s multiple range tests. The differences between the two groups were assessed using *t*-tests at a significance level of *p* < 0.05.

## 5. Conclusions

In the current study, we demonstrate the reduction in oxidative and melanogenic activities in melan-a cells by the mixture of resveratrol- and PPD-enriched rice seed extracts. TR_3 containing 70% (*w*/*w*) of resveratrol-enriched rice seed extract and 30% (*w*/*w*) of PPD-enriched rice seed extract exhibited antioxidant effects by inhibiting ABTS radical density, which was similar to those of DJ526 and DJ_PPD alone. The mRNA expression level of MITF was significantly downregulated in TR_3-treated cells when compared to DJ-PPD. TR_3 remarkedly suppressed the production of MITF protein compared to DJ526- and DJ-PPD-treated cells alone. The reduction in MITF by TR_3 led to the downregulation of TRP-1, TRP-2, and tyrosinase, both mRNA and protein levels. TR_3 enhanced the production of p-ERK 1/2 and p-Akt proteins and decreased the p-p38 MAPK production. These regulations of melanogenesis-associated biomarkers result in the inhibition of melanin content and cellular tyrosinase activity in melan-a cells. Additionally, the morphological appearance analysis showed that TR_3 reduced the number of melanin-containing cells, size, and melanin dispersion in melan-a cells. Overall, the results signify that the combination of the two compounds could significantly trigger morphological and melanogenic synthesis pathway control, causing a decrease in melanin production.

Therefore, we suggest that the combination of resveratrol- and PPD-enriched rice seed extracts could be used as a potential agent for depigmentation. Moreover, the results set a new perspective in the field of nutraceutical formulation parameters on combining compounds to achieve synergism that can cause better results at lower dosage, thus conserving biological resources available.

## Figures and Tables

**Table 1:** Vitamin C equivalent antioxidant capacity and IC_50_ of each treatment.

Treatment	Concentration (mg/mL)	IC_50_		VCEAC: mg/g	
		Mean	Standard Deviation	Mean	Standard Deviation
DMSO	0.10%	−	−	0.00	0.003
DJ526	10	40.75 ^c^	0.66	0.044	0.003
	25			0.095	0.003
	50			0.144	0.006
	100			0.234	0.002
DJ-PPD	10	40.11 ^c^	0.59	0.061	0.002
	25			0.088	0.005
	50			0.135	0.002
	100			0.236	0.001
TR_1	10	67.06 ^a^	1.36	0.019	0.007
	25			0.055	0.004
	50			0.103	0.003
	100			0.164	0.004
TR_2	10	56.65 ^b^	2.78	0.023	0.004
	25			0.068	0.002
	50			0.121	0.005
	100			0.187	0.009
TR_3	10	43.52 ^c^	0.29	0.043	0.004
	25			0.091	0.004
	50			0.128	0.002
	100			0.233	0.001

VCEAC: Vitamin C equivalent antioxidant capacity. Lowercase letters represent the significant difference level of IC_50_ between the treatments at *p* < 0.05. The letter “a” represents the reference value, “b” represents significantly lower value than “a” (*p* < 0.05), and “c” represents significantly lower value than “b” (*p* < 0.05).

**Table 2:** Total RNA quantity and quality.

Treatment	Quantity of Total RNA (ng/µL)			Quality of Total RNA	
	Mean	Standard Deviation	Coefficient of Variation	A260:A280	A260:A230
Medium	354.83	7.40	2.09	1.972	1.894
DMSO	380.23	9.77	2.57	1.931	1.855
Arbutin	358.46	22.85	6.37	1.980	1.920
DJ526	430.33	12.74	2.96	1.943	1.911
DJ-PPD	384.32	10.78	2.81	1.925	1.922
TR_1	459.27	20.40	4.44	1.975	1.908
TR_2	470.47	14.25	3.03	1.982	1.917
TR_3	650.01	21.07	3.24	1.946	1.868

**Table 3:** Antibodies used for the Western blot analysis.

Primary Antibody	Company	Dilution (*v*/*v*)	Secondary Antibody	Company	Dilution (*v*/*v*)
MITF	Santa Cruz Biotechnology	1:1000	m-IgGκ BP-HRP	Santa Cruz Biotechnology	1:5000
TRP-1	Santa Cruz Biotechnology	1:1000	m-IgGκ BP-HRP	Santa Cruz Biotechnology	1:5000
TRP-2	Santa Cruz Biotechnology	1:1000	m-IgGκ BP-HRP	Santa Cruz Biotechnology	1:5000
Tyrosinase	Santa Cruz Biotechnology	1:1000	m-IgGκ BP-HRP	Santa Cruz Biotechnology	1:5000
p-ERK 1/2	Cell Signaling	1:2000	Goat anti-rabbit IgG (H + L)-HRP	GenDEPOT	1:5000
p-Akt	Cell Signaling	1:2000	Goat anti-rabbit IgG (H + L)-HRP	GenDEPOT	1:5000
p-p38 MAPK	Cell Signaling	1:2000	Goat anti-rabbit IgG (H + L)-HRP	GenDEPOT	1:5000
ERK 1/2	Cell Signaling	1:2000	Goat anti-rabbit IgG (H + L)-HRP	GenDEPOT	1:5000
Akt	Santa Cruz Biotechnology	1:1000	m-IgGκ BP-HRP	Santa Cruz Biotechnology	1:5000
p38 MAPK	Santa Cruz Biotechnology	1:1000	m-IgGκ BP-HRP	Santa Cruz Biotechnology	1:5000
GAPDH	Santa Cruz Biotechnology	1:2000	m-IgGκ BP-HRP	Santa Cruz Biotechnology	1:5000

Note: Santa Cruz Biotechnology (Dallas, TX, USA). Cell Signaling (Danvers, MA, USA). GenDEPOT (Baker, TX, USA). The antibody was diluted in 1× TBST supplemented with 5% (*w*/*v*) skim milk (antibody:diluent).

## Data Availability

All applicable data have been provided in the manuscript. The authors will provide additional details if necessary.

## Supplementary Materials

The following supporting information can be downloaded at: https://www.mdpi.com/article/10.3390/plants13233385/s1, Figure S1: Standard curve of the radical scavenging activity of ABTS plotted against the varied treatment concentrations; Figure S2: Standard curve of the radical scavenging activity of ABTS plotted against the varied concentrations of ascorbic acid (vitamin C); Figure S3: Morphological appearance of the differentiated melan-a cell scoring; Table S1: Piceid, resveratrol, and PPD content in the study treatment; Table S2: Differentiated melan-a cell scoring.
