# Supplementary material for: Coinhibitory Effects of Resveratrol- and Protopanaxadiol-Enriched Rice Seed Extracts Against Melanogenic Activities in Melan-a Cells
Source: Plants (Basel). 2024 Dec 1;13(23):3385. doi: 10.3390/plants13233385 (PMC11644661; doi:10.3390/plants13233385)
Supplement: Supplementary file 1 [file plants-13-03385-s001.zip › plants-3303102-supplementary.pdf]

Supplementary materials

Figure S1. Standard curve of the radical scavenging activity of ABTS plotted against the varied treatment concentrations

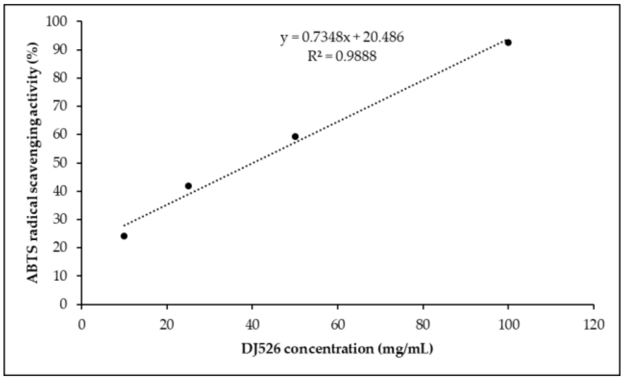

DJ526

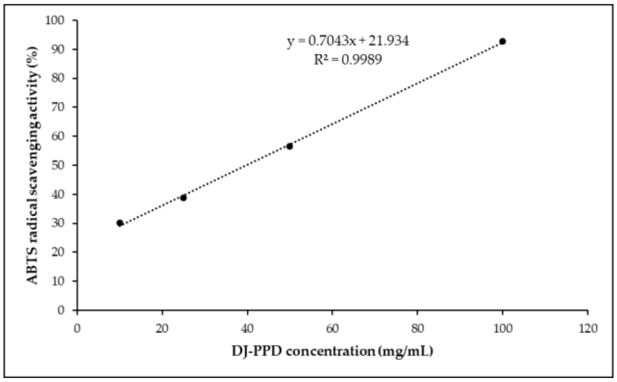

DJ-PPD

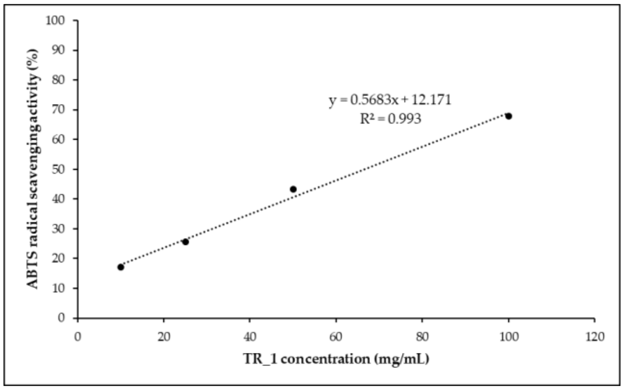

TR_1

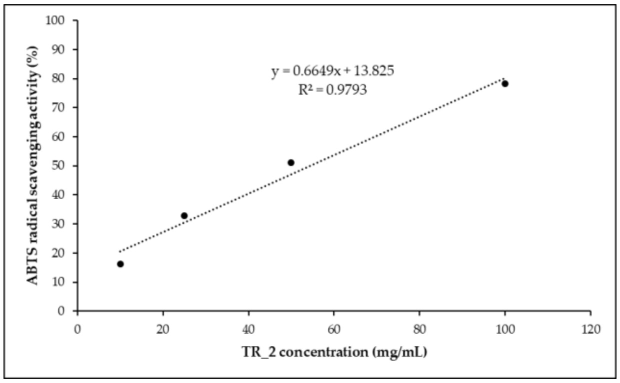

TR_2

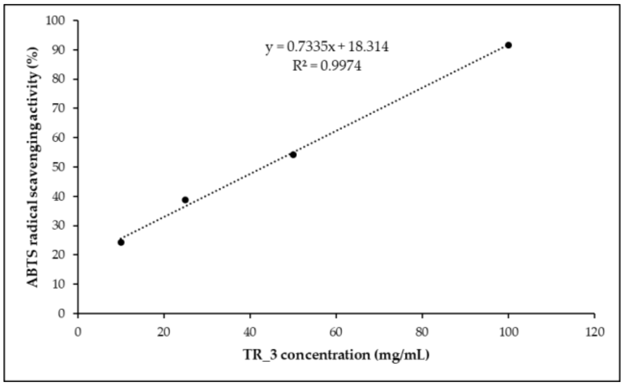

TR_3

Figure S2. Standard curve of the radical scavenging activity of ABTS plotted against the varied concentrations of ascorbic acid (vitamin C)

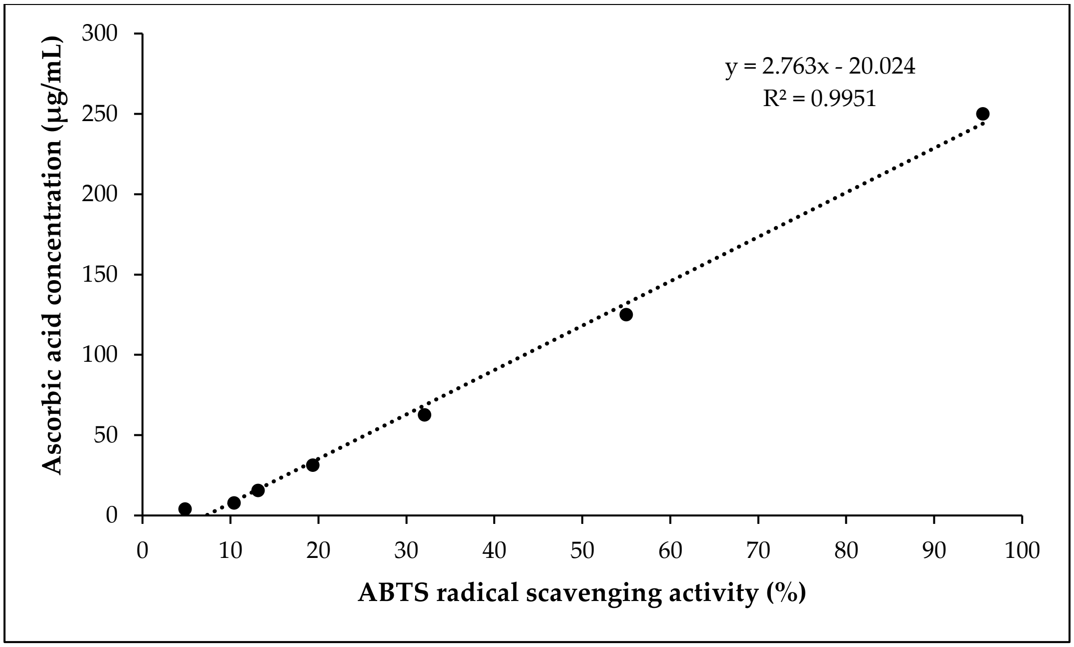

Figure S3. Morphological appearance of the differentiated melan-a cell scoring

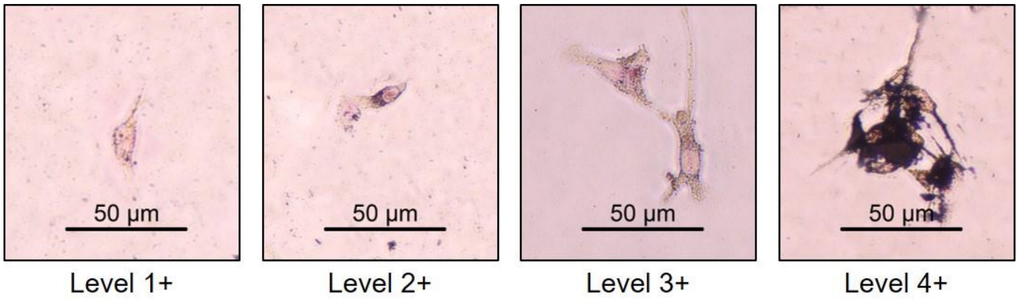

Table S1. Piceid, resveratrol, and PPD content in the study treatment

Treatment	DJ526 (% *w/w*)	DJ-PPD (% *w/w*)	Piceid content (×10⁻² ng)	Resveratrol content (×10⁻² ng)	PPD content (×10⁻² ng)
DJ526	100	0	47.2	26.1	0.0
DJ-PPD	0	100	0.0	0.0	72.8
TR_1	30	70	14.2	7.8	51.0
TR_2	50	50	23.6	13.1	36.4
TR_3	70	30	33.0	18.3	21.8

Table S2. Differentiated melan-a cell scoring

**Benchmark**	**Score**

Cell size <50 µm and pigmentation <50% of the cytoplasmic area with a low distribution	1+
Cell size <50 µm and pigmentation equal or >50% of the cytoplasmic area with a higher distribution	2+
Cell size >51 µm and pigmentation throughout the cytoplasmic area	3+
Cell size >51 µm and pigmentation densely packed throughout the cytoplasmic area	4+
